# A Novel Bocavirus Associated with Acute Gastroenteritis in Australian Children

**DOI:** 10.1371/journal.ppat.1000391

**Published:** 2009-04-17

**Authors:** Jane L. Arthur, Geoffrey D. Higgins, Geoffrey P. Davidson, Rodney C. Givney, Rodney M. Ratcliff

**Affiliations:** 1 Infectious Diseases Laboratories, Institute of Medical and Veterinary Science, Adelaide, Australia; 2 School of Molecular & Biomedical Science, University of Adelaide, Adelaide, Australia; 3 Gastroenterology Unit, Women's and Children's Hospital, North Adelaide, Australia; 4 Department of Pediatrics, University of Adelaide, Adelaide, Australia; 5 Microbiology, Hunter Area Pathology Service, Newcastle, Australia; Brigham and Women's Hospital/Harvard Medical School, United States of America

## Abstract

Acute gastroenteritis (AGE) is a common illness affecting all age groups worldwide, causing an estimated three million deaths annually. Viruses such as rotavirus, adenovirus, and caliciviruses are a major cause of AGE, but in many patients a causal agent cannot be found despite extensive diagnostic testing. Proposing that novel viruses are the reason for this diagnostic gap, we used molecular screening to investigate a cluster of undiagnosed cases that were part of a larger case control study into the etiology of pediatric AGE. Degenerate oligonucleotide primed (DOP) PCR was used to non-specifically amplify viral DNA from fecal specimens. The amplified DNA was then cloned and sequenced for analysis. A novel virus was detected. Elucidation and analysis of the genome indicates it is a member of the Bocavirus genus of the Parvovirinae, 23% variant at the nucleotide level from its closest formally recognized relative, the Human Bocavirus (HBoV), and similar to the very recently proposed second species of Bocavirus (HBoV2). Fecal samples collected from case control pairs during 2001 for the AGE study were tested with a bocavirus-specific PCR, and HBoV2 (sequence confirmed) was detected in 32 of 186 cases with AGE (prevalence 17.2%) compared with only 15 controls (8.1%). In this same group of children, HBoV2 prevalence was exceeded only by rotavirus (39.2%) and astrovirus (21.5%) and was more prevalent than norovirus genogroup 2 (13.4%) and adenovirus (4.8%). In a univariate analysis of the matched pairs (McNemar's Test), the odds ratio for the association of AGE with HBoV2 infection was 2.6 (95% confidence interval 1.2–5.7); P = 0.007. During the course of this screening, a second novel bocavirus was detected which we have designated HBoV species 3 (HBoV3). The prevalence of HBoV3 was low (2.7%), and it was not associated with AGE. HBoV2 and HBoV3 are newly discovered bocaviruses, of which HBoV2 is the thirdmost-prevalent virus, after rotavirus and astrovirus, associated with pediatric AGE in this study.

## Author Summary

Acute gastroenteritis (AGE) is a common illness affecting all age groups worldwide, causing an estimated three million deaths annually. However, in many patients a causal agent cannot be found despite extensive diagnostic testing. Proposing that novel viruses are the reason for this diagnostic gap, we screened fecal samples from symptomatic children using a molecular degenerate amplification technique and detected the presence of a novel parvovirus, Human Bocavirus species 2 (HBoV2). The genome of HBoV2 is 23% variant from its closest relative, the human bocavirus, a member of the Bocavirus genus of the Parvovirinae. Using specific amplification assays, we then found HBoV2 was the thirdmost-prevalent virus detected in samples from symptomatic children in a case control study of AGE. Further, we found virus presence was associated with symptoms. During this screening, we detected a second related parvovirus, which we have named Human Bocavirus species 3 (HBoV3), but the prevalence was low and not associated with symptoms. The discovery of HBoV2 has reduced the diagnostic gap, but more studies are required to further investigate its role in AGE.

## Introduction

Acute gastroenteritis (AGE) is a common illness affecting all age groups worldwide. Even in developed countries, most of the population will average one episode of AGE per year [1]. Among young children and the elderly, the rate is higher and the disease more severe [2],[3]. Globally, approximately 1.5 billion episodes and 1.5 to 2.5 million deaths annually in children under age five are estimated to be associated with AGE, the majority occurring in developing countries [4]. Among the elderly, underlying immunological, physiological and functional deterioration exacerbate severity, resulting in increased morbidity and mortality attributed to diarrheal disease. Mortality may exceed 50% in the above 73 years age group in the US [3]. However, for such an important disease, our understanding of causality remains incomplete. The importance of bacteria and parasites in AGE has been known for many years, although the roles of campylobacter (1972) [5] and cryptosporidium (1976) [6] in human infection were recognized more recently. Viruses such as rotavirus and enteric adenovirus were also first described over 30 years ago and shown to have an association with diarrheal disease. Rotavirus is the most common cause of viral AGE in children under age five [7]. The recent widespread availability of molecular diagnostics has allowed an increased understanding of the role of other viruses in causing AGE. The human calicivirus genus Norovirus (NoV, formerly Norwalk-like virus, NLV) is now recognized as the most common cause of epidemic outbreaks of AGE across all age groups [1] with noroviruses belonging to genogroup 2 (NoV GII) more frequent than those belonging to genogroup 1 (NoV GI). Recent reports suggest prevalence rates of 9 to 20% for NoV in sporadic AGE in children [8]–[11]. Similarly, associations with AGE have also been made for both a second calicivirus genus Sapovirus (formerly Sapporo-like virus, SLV or classic calicivirus) and astrovirus. A limited number of reports suggest sapoviruses are less frequently found in cases of AGE than NoVs, are mainly restricted to children less than age five years and may cause less severe symptoms than infections with rotavirus and NoV [12]–[14]. Human astroviruses are reported to have an association with AGE, especially in children, with prevalence rates up to 8.6% documented [15]–[17]. Higher rates (39%) have been reported in children less than 1 year old [18] and in areas lacking adequate sanitation [19]. Other viruses such as torovirus, picobirnavirus, picotrinavirus, pestivirus and coronavirus have also been implicated [20]–[23]. Lately, there have been reports of human bocavirus (HBoV) sequences in feces of children with respiratory infections or AGE [24]–[26]. However, many of these observations are from uncontrolled trials and in the absence of large prospective controlled studies, their association with AGE is uncertain. Despite this wide range of known and suspected pathogens, in both pediatric and adult populations, the pathogen causing AGE remains unidentified in up to 67% of cases [7],[14],[27],[28].

In order to further investigate the causes of AGE in children, in late 2000 we commenced a prospective controlled study of patients presenting to a children's hospital in Adelaide, South Australia. Unexpectedly, even with extensive molecular testing, we could not find a cause in 28% of cases (Ratcliff, R.M. unpublished). Proposing that novel viruses may be the cause of the undiagnosed cases, we developed a method for ‘non-selective’ detection of viral DNA in feces. This method uses degenerate oligonucleotide primed (DOP) PCR amplification [29] to non-specifically amplify DNA from diarrheal samples. Previously, DOP amplification had been utilized to replicate DNA from minute samples such as single cell nuclei, and forensic and archaeological sources [29]. An alternative ‘non-specific’ amplification method used nuclease digestion to specifically purify virus nucleic acid by exploiting the protection of the virus nucleic acid from digestion afforded by the virus capsid [30]. Combining nuclease digestion with DOP amplification ought to facilitate the sole replication of viral nucleic acid from nucleic acid ‘soups’ such as feces. The DNA produced from such virus enrichment followed by non-specific amplification can be cloned and sequenced to assess the presence of viral DNA and provide a method to screen for novel enteric viruses. We initially investigated a ‘summer’ cluster of sporadic AGE cases occurring between January and April 2001 and who were part of the case control study into the etiology of pediatric AGE. We detected a previously unknown parvovirus, which we have named Human Bocavirus species 2 (HBoV2). Following the development of specific amplification assays to detect HBoV2, we screened all samples collected from cases and controls in the study during 2001 and assessed their association with pediatric AGE. During this screening, we discovered a second novel parvovirus which we have named Human Bocavirus species 3 (HBoV3).

## Results

### Routine screening for known causal agents

Samples from 197 cases and 197 aged-matched controls were available for analysis. A bacterial pathogen was isolated in only 9 patients in the first sample tested, with one additional salmonella isolate and one yersinia isolated in second samples from another two patients. In total, only 11 patients had bacterial pathogens identified. No bacterial testing was done on the controls. A ‘known’ viral pathogen (NoV GI, NoV GII, sapovirus, astrovirus, rotavirus or adenovirus) was found in first or second specimens in 125/197 (63.5%) of cases and in 49/197 (24.9%) of controls. Overall a ‘known’ viral or bacterial pathogen was found in 134/197 (68.0%) of cases (2 salmonella positive cases were also rotavirus infected, one of whom also had adenovirus). Therefore 32% of AGE cases remained without a diagnosis.

### Discovery of novel virus sequence

In February 2001, a 9 month old male presenting with coryza, fever, vomiting and diarrhea was enrolled in the case control study and fecal samples collected at the time of presentation (W153) and 3 days later (W154). No microbial or virological agent associated with AGE was initially detected in either sample despite extensive testing. As one of a number of ‘pathogen negative’ samples being screened, the viral nucleic acid fraction of the fecal suspension was prepared, DOP amplified and products examined by cloning and sequencing. A total of 43 PCR products were sequenced from the initial sample (W153) and GenBank homology searching (tBLASTx) indicated 38 showed between 46% and 91% amino acid homology to the human bocavirus (HBoV). A further 31 PCR products derived from sample W154 were sequenced and 24 showed amino acid homology of between 42% and 91% to HBoV. The remaining sequences showed no evidence of significant homology to known viruses.

Using the HBoV strain st2 (GenBank DQ000496) as a reference, the 55 DOP-generated sequences were aligned and many overlapped to form a discontinuous sequence of approximately 3300 nucleotides of the new virus genome (>60%) that appeared to lack at least 1000 and 750 nucleotides from the left and right ends of the genome respectively. PCR primers homologous to the DOP-generated sequences were designed to amplify and sequence products from sample W153 to span the sequence gaps and similarly the semi-DOP PCR approach was employed to extend the virus sequence in both up and downstream directions. For sequence confirmation and to remove potential errors generated during the DOP amplification, the sequence was rederived using primers homologous to the DOP-generated sequence in a series of specific PCR reactions to amplify and sequence overlapping products directly from sample W153. Each nucleotide was confirmed by a minimum of two sequences from separate PCR reactions for all 5204 nucleotides of the sequence. The sequence of similarly generated product from the second sample from the same patient, W154, was homologous to W153 sequence.

### Genome sequence analysis of W153

**Figure 1:**
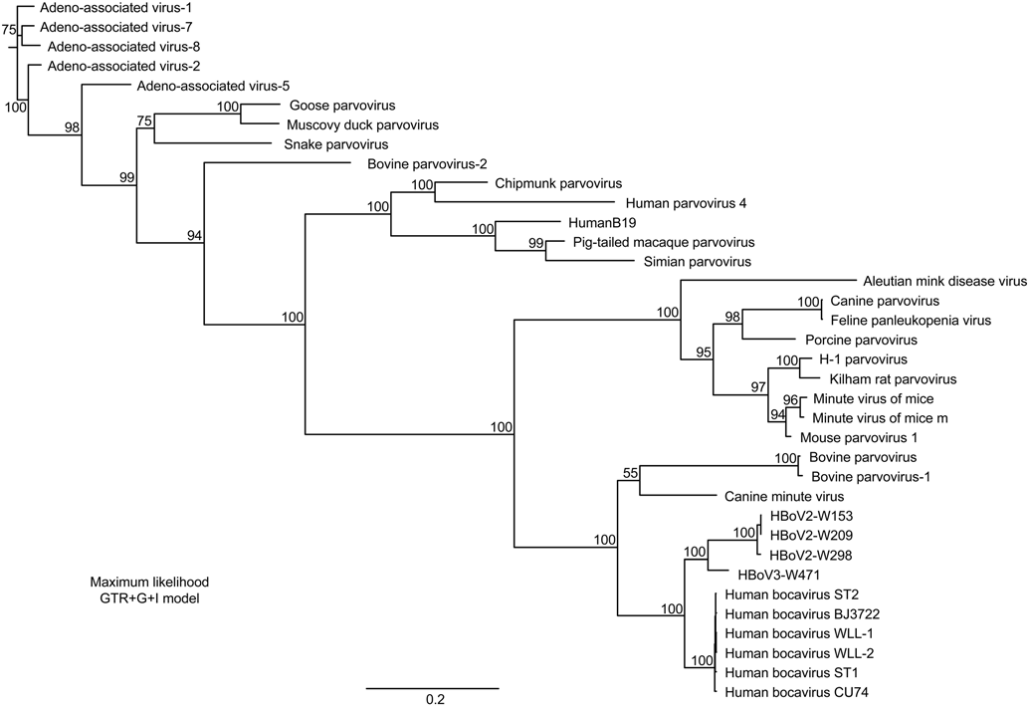
Phylogenetic analysis of parvoviruses: Maximum likelihood dendrogram (bootstrapped, 1,000 replicates) based on full-length parvovirus genome sequence (nucleotide) alignments. Approximately 100–200 nucleotides are absent from each termini of the HBoV2, HBoV3, and HBoV sequences.

**Figure 2:**
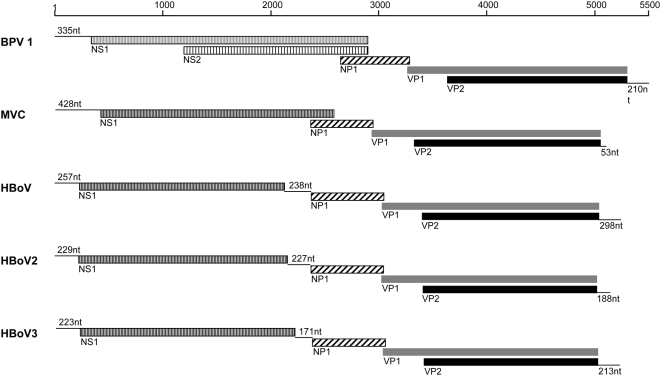
The HBoV2 and HBoV3 genomes. Diagrammatic representation of HBoV2 (HBoV2-W153, 5204 nucleotides) and HBoV3 (HBoV3-W471, 5242 nucleotides) sequence showing the position of open reading frames for NS1, NP1, VP1, and VP2, compared with other bocaviruses: bovine parvovirus 1 (BPV 1), minute virus of canines (MVC), and human bocavirus (HBoV). For both HBoV2 and HBoV3, as with HBoV, the NP1 gene is in an alternate reading frame to VP1 and overlaps the start of VP1 by 13 nucleotides. Similarly, VP2 is collinear to VP1 and results from initiation of translation at a downstream ATG and co-terminates.

Homology alignments with selected members of the Parvovirinae, including all members of the genus Bocavirus (bovine parvovirus 1, minute virus of canines and HBoV), confirm that W153 is a member of the Bocavirus genus of the Parvovirinae (Figure 1). From this alignment we predict that the W153 sequence spans the entire coding region of the virus genome but lacks up to 100 nucleotides of both terminal regions. At the nucleotide level the W153 sequence is 23% variant from its closest relative, the HBoV but contains open reading frames (ORFs) for NS1, NP1, VP1 and VP2 proteins in a similar arrangement to the HBoV including the alignment of the NP1 ORF in an alternate reading frame to VP1 whereby it overlaps the start of the VP1 by 13 nucleotides (Figure 2). A comparison of the length of the coding regions, and the genetic distance (percent dissimilarity) between them, for W153 and HBoV at the nucleotide and amino acid level are presented in Table 1. These results are in contrast to strains of the HBoV where the homology between all strains exceeds 98% at both nucleotide and amino acid level for all ORFs [24],[31]. The ICTVb criteria for defining a new species in the bocavirus genus is a non-structural gene genetic homology of less than 95% (C. Büchen-Osmond, International Committee on Taxonomy of Viruses, http://www.ncbi.nlm.nih.gov/ICTVdb/Ictv/fs_parvo.htm, Columbia University, New York, USA). Thus we propose that the virus detected in sample W153 is not a new strain of the HBoV but rather a new species of Bocavirus (family Parvoviridae subfamily Parvovirinae) and have assigned the name Human Bocavirus species 2 (HBoV2; see Discussion and reference 35 for further details). The sequence of the HBoV2-W153 strain has been deposited in GenBank (designated HBoV2-W153, accession number EU082213).

**Table 1:** Comparison of the open reading frames for HBoV2-W153, HBoV2-W208, HBoV3-W471, and HBoV st1.

	Virus or Strain	NS1	NP1	VP1	VP2
**Amino acids** *	HBoV2	641	216	668	539
	HBoV3	662	219	669	540
	HBoV	640	220	672	543
**Pairwise dissimilarity** †	HBoV2-W153 to HBoV2-W208	0.05 (0)	0.3 (0)	0.05 (0)	0.06 (0)
	HBoV2-W153 to HBoV st1	25.8 (22.4)	22.7 (29.8)	21.2 (19.3)	23.1 (21.6)
	HBoV2-W153 to HBoV3-W471	24.2 (23.1)	21.2 (29.7)	12.3 (9.0)	14.2 (10.4)
	HBoV3-W471 to HBoV st1	12.7 (8.9)	13.2 (16.5)	23.3 (19.5)	24.6 (21.7)

*: Number of amino acids encoded for each protein.

**†:** Pairwise percent dissimilarity at both the nucleotide and amino acid (parentheses) levels.

### Discovery of a second novel bocavirus while assessing the prevalence of HBoV2 in children

**Figure 3:**
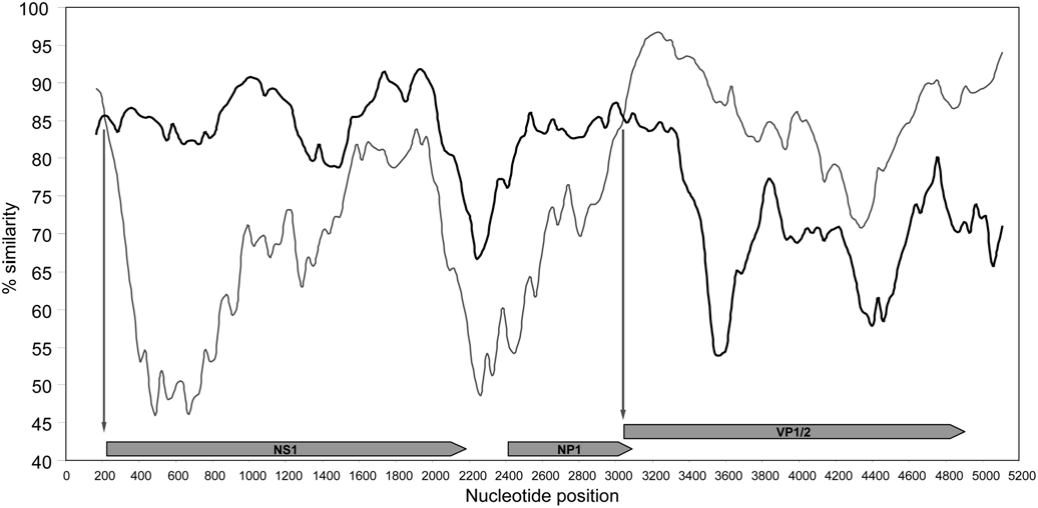
Genome similarity plot of HBoV3-W471 against HBoV (black line, DQ000495) and HBoV2-W153 (grey line) showing percentage similarity along the aligned genomes (SimPlot 3.2, 2-parameter (Kimura) distance model, window size is 300 bp, step size is 30 bp). Approximate recombination sites are indicated by the crossover of the sequence most similar to HBoV3-W471 at positions 200 and 3,000 nucleotides, just upstream of NS1 and VP1/2 ORFs; highlighted by the two arrows. Note the high level of conservation between all three genomes toward the 3′ terminus of NS1 and the 5′ terminus of VP1/2 ORFs, presumably the result of functional constraint.

To examine whether HBoV2 was associated with AGE we used the nested consensus PCR assay to screen fecal samples from the 197 matched case-control pairs, confirming all positives by sequencing. The sequence analysis showed the presence of both HBoV2 and HBoV, as well as another novel parvovirus. The genome of this virus from sample W471 was sequenced using specific PCRs incorporating primers homologous to recovered W471 sequence and homologous regions in an alignment of HBoV2-W153 and HBoV, plus a semi-DOP approach similar to that used for HBoV2-W153 to extend the sequence, to recover 5242 nucleotides of sequence spanning the entire coding region, but missing the termini sequences as for HBoV2-W153. The alignment of the W471 sequence with other Parvovirinae confirms it is also a bocavirus (Figure 1) with the genetic distance of the ORFs at both nucleotide and amino acid level (Table 1) indicative it is a novel bocavirus species. To the virus in sample W471, we have assigned the name Human Bocavirus species 3 (HBoV3; GenBank EU918736). The unusual pattern of similarity for HBoV3 seen in Table 1 (genetically closer to HBoV in the non-structural protein-encoding regions but to HBoV2 in the structural protein-encoding region) suggests that HBoV3 might be the result of an ancestral recombination event between HBoV and HBoV2. To investigate this possibility a similarity analysis (Simplot) was performed comparing HBoV3 with HBoV and HBov2. This confirmed that two recombination sites are present, immediately upstream of both NS1 and VP1/2 ORFs (Figure 3).

### Virus prevalence in children

The analysis of virus prevalence in samples collected during 2001 is presented in Table 2. Since bacterial isolation was only performed on cases we have excluded from the statistical analysis the 11 cases with bacterial pathogens (of which one was HBoV2 positive, one HBoV2 and astrovirus, one astrovirus and adenovirus and one HBoV) and their associated 11 controls (of which 3 were HBoV2 positive, one HBoV2 plus HBoV and one astrovirus positive). Thus 186 matched case control pairs were used for the statistical analysis of the association of viruses with disease. The mean age of the 186 cases was 2.7 years, median age 1.7 years, age range 1 day to 17.6 years. One hundred and twenty-two of the cases were male. The mean age of the controls was 2.6 years, median 1.8 years, age range 1 day to 16.5 years. One hundred and twenty two of the controls were male.

**Table 2:** Univariate analysis of viruses detected in matched case control pairs enrolled during 2001.

		HBoV2	HBoV3	Rotavirus	Astrovirus	NoV GII	HBoV	Adenovirus	Sapovirus	NoV GI	Total Patients Positive	Total Patients with Multiple Viruses
**Single sample**	Case*	22	5	69	33	22	16	7	5	1	134 (72%)	37§ (19.9%)
	Control*	15	4	2	22	15	11	2	6	5	68 (36.6%)	15∥ (8%)
	P value	0.2	0.7	<0.0001	0.1	0.2	0.3	0.06	0.7	0.1		
	OR† (CI‡)	1.6 (0.7–3.6)	1.3 (0.3–6.3)	68 (11.8–2725)	1.7 (0.9–3.4)	1.5 (0.7–3.2)	1.5 (0.6–3.7)	6 (0.7–276)	0.8 (0.2–3.7)	0.2 (0.004–1.8)		
**Including additional samples from cases within 3 days**	Case*	32	5	73	40	25	17	9	6	1		
	Control*	15	4	2	22	15	11	2	6	5	141 (75.8%)	51 (27.4%)
	P value	0.007	0.7	<0.0001	0.01	0.1	0.2	0.02	1.0	0.1	68 (36.6%)	15 (8%)
	OR† (CI‡)	2.6 (1.2–5.7)	1.3 (0.3–6.3)	72 (12.5–2883)	2.4 (1.2–5.0)	1.7 (0.9–3.6)	1.6 (0.7–3.9)	1.7 (1.1–355)	1.0 (0.2–4.4)	0.2 (0.004–1.8)		

Two-tailed univariate analysis by McNemar's test for correlated proportions comparing matched case-control pairs concordant and discordant for exposure to an individual virus.

*: Viruses detected from 186 cases and 186 age-matched paired controls in which recognized bacterial pathogens were not detected.

**†:** OR, odds ratio.

**‡:** CI, 95% confidence interval.

**§:** Multiple viruses in 20 different combinations.

**∥:** Multiple viruses in 7 different combinations.

Only one sample was collected from each control whereas 105 of the 186 cases had a second sample collected within 3 days. Thus the univariant analysis of matched case control pairs was performed with and without the inclusion of the second sample from cases. When including only a single (first) sample from each case, the univariant analysis showed an association between infection and AGE only for rotavirus, which was also the most prevalent virus detected (Table 2). When including the second case samples in the analysis, an association between infection and AGE was additionally found for HBoV2, adenovirus and astrovirus. Because of the high frequency of multiple viruses detected in samples from cases (51, 27.4%) and controls (15, 8%), a multivariate conditional logistic regression analysis was performed on the pooled case data using all detections which had an association P<0.2 in univariate analysis (Table 3). In this analysis, an association between infection and AGE was also found for NoV GII in addition to those associated in the pooled univariant analysis; rotavirus, HBoV2, adenovirus and astrovirus.

**Table 3:** Conditional logistic regression of all viruses associated with gastroenteritis with P value<0.2 in the univariate analysis (Table 2, pooled samples from cases).

Virus Detected*	P Value	OR† (CI) ‡
HBoV2	0.03	2.9 (1.1–7.7)
Rotavirus	<0.001	92.4 (12.2–702)
Astrovirus	0.008	3.6 (1.4–9.0)
Norovirus GII	0.006	4.1 (1.5–11.1)
Adenovirus	0.03	12.2 (1.3–111)
Norovirus GI	0.6	0.5 (0.05–5.7)

*: Viruses detected from 186 cases and 186 age-matched paired controls in which recognized bacterial pathogens were not detected.

**†:** OR, odds ratio.

**‡:** CI, 95% confidence interval.

In the complete data set, samples from the 197 case control pairs, a pathogen could not be detected in 63 of cases (32.0%) prior to screening for HBoV2. HBoV2 was detected in 11 of these ‘pathogen negative’ cases, a reduction of cases to 52 (26.4%) which represents a 17.5% reduction in the diagnostic gap in this data set.

Although we originally targeted a ‘summer’ peak of AGE cases to screen for the presence of novel viruses, HBoV2 was detected throughout 2001, predominantly between January and mid-September (in the Southern Hemisphere, mid-summer to early spring).

### Sequence analysis of bocavirus strains: partial NS1 sequence

**Figure 4:**
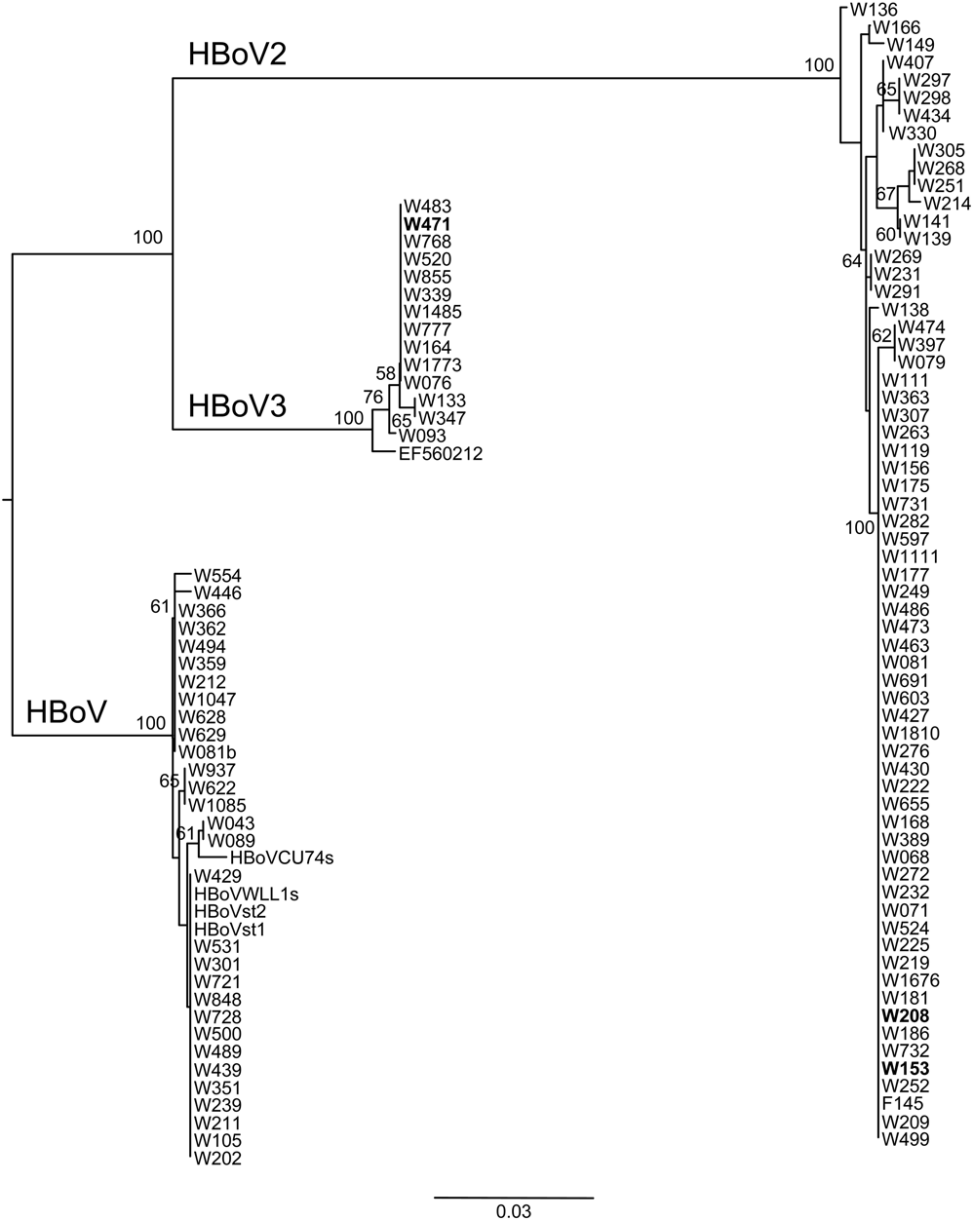
Maximum likelihood dendrogram (bootstrapped, 1,000 replicates) of HBoV, HBoV2, and HBoV3 partial NS1 sequences.

The partial NS1 sequences from all HBoV, HBoV2 and HBoV3 strains detected during this study were aligned and phylogenetically compared (Figure 4). All show a high degree of nucleotide conservation within each of the three clades HBoV, HBoV2 and HBoV3, similar to the recent observations for HBoV [24],[31]. The virus sequence from sample W208, designated HBoV2-W208, was extended using the same primer sets as used to rederive HBoV2-W153, to recover 5156 nucleotides (99.9% similar to HBoV2-W153) encompassing the complete encoding region but also missing the termini (GenBank accession EU082214).

### Detection of HBoV in diarrhea

Recently, there has been reports of detecting HBoV sequences in feces associated with respiratory infections, or AGE, in children [24],[26]. Although these observations are largely from uncontrolled specimens, we assessed the prevalence of HBoV in our data from matched case control pairs. In addition, samples were also screened using PCR incorporating published HBoV primers [32] but no additional positives were detected. In total, HBoV was detected in 17 case samples (9.1%) compared with 11 controls (5.9%). HBoV was not statistically associated with AGE.

## Discussion

This study reports the discovery of two new parvoviruses, HBoV2 and HBoV3. Based on a comparison with other species, the 5204 and 5242 nucleotide sequences recovered for these virus respectively, represents over 98% of the predicted genome for both viruses, each containing the ORFs typical of other bocaviruses and only lacking both termini. Our initial attempts to sequence the termini have failed, consistent with Allander's observations that the terminal regions of HBoV are problematic [32], most likely due to hairpin loop structures as identified at the termini of other parvoviruses [33]. Work to clarify the sequence of the viral termini is ongoing. Genetic homology alignments place both HBoV2 and HBoV3 in the Bocavirus genus of the Parvovirinae (Figure 1) and both have a similar genome arrangement of putative ORFs to HBoV. HBoV2 is 23% variant from HBoV, its closest previously known relative. HBoV3 lies between the two, 18% variant both from HBoV2 and HBoV at the genome level. The level of dissimilarity to HBoV is maintained for HBoV2 across the coding regions at both nucleotide and amino acid levels. Intriguingly, this is not the case for HBoV3, where the non-structural ORFs NS1 and NP1 are more similar to HBoV, where as the structural ORF (VP1/VP2) is more similar to HBoV2 (Table 1). Thus HBoV3 may be the consequence of a recombination event between ancestral forms of HBoV2 and HBoV. The SimPlot analysis on an alignment of the three viral genomes (Figure 3) supports this hypothesis, indicating recombination sites close to genome positions 200 and 3000, just upstream of the NS1 and VP1/2 ORFs. There are homologous regions in both these locations which would facilitate such an event. Interestingly, recombination events have been reported for NoV GII with the recombination site similarly located in conserved sequence immediately upstream of the capsid-encoding ORF [34]. Nevertheless, since there is less than 95% genetic similarity for all putative ORFs including those encoding non-structural proteins (NS1 and NP1), this is consistent with both HBoV2 and HBoV3 being new species of bocavirus.

Very recently, Kapoor *et al.* [35] reported the presence of a novel Bocavirus. An analysis of the sequence (FJ170280) confirms their novel bocavirus is very similar (>99.7%) to HBoV2-W153, indicating that the same virus has been simultaneously discovered by two groups. Further, since they detected HBoV2 in samples from children in Pakistan and Scotland, the combined reports infer HBoV2 is globally distributed and likely to be commonly associated with pediatric AGE in every community.

The family Parvoviridae are among the smallest viruses measuring 18 to 26 nm, unenveloped, with an icosahedral capsid. Their genomes are single, mostly negative-stranded linear DNA between 2 and 6 kb in length [36]. Other parvoviruses, such as canine parvovirus and feline panleukopenia virus, are associated with gastroenteritis or enteric disease of their natural host [36] and it was perhaps anomalous that to date there had not been a parvovirus with a similar association in humans. In addition, other parvoviruses are known to replicate only when the host cell is in S phase, and results in the death of the host cell [36]. This would suggest that virus shedding may peak later in the course of an infection as any damaged and dead cells are replaced.

Our analysis of the prevalence and association with AGE is based on samples from 186 age-matched case control pairs collected during 2001 as part of a larger prospective study of over 600 cases and matched controls. In the univariant analysis of first samples, only rotavirus infection was significantly associated with AGE (Table 2). The inclusion of second samples collected within three days of presentation for 105 cases resulted in significant AGE-association for HBoV2, adenovirus and astrovirus, in addition to rotavirus. It was only in the multivariate conditional logistic regression analysis (Table 3) that NoV GII was associated with AGE. Thus, although an association with AGE was not achieved for HBoV2 in the most rigorous univariant analysis so further conclusions require caution until additional data are available, it is persuasive that an association with AGE was achieved for HBoV2 in the same data analysis (pooled case samples) in which significance was achieved for adenovirus and NoV GII, the role of which as pathogens causing AGE is not disputed [37]. HBoV2, at 17.2%, was the third most prevalent virus after rotavirus (39.2%) and astrovirus (21.5%), and was greater than that for NoV GII (13.4%) and adenovirus (4.8%). Two further factors may be influencing the prevalence rates for HBoV2 we have detected in both cases and controls. Firstly, S phase synchronized replication observed for other parvoviruses may also be applicable for HBoV2, in which case delayed and perhaps cyclical or pulsed shedding of HBoV2 during infection may result, consistent with our finding that 10 cases (31.2% of positive cases) had detectable virus only in their second sample. Therefore, the statistical association in the pooled sample data may better reflect the true disease association. Secondly, long term virus shedding post-infection, which is increasingly being recognized for other enteric viruses such as NoV GII [38], may also account for asymptomatic carriage among the controls. However, until the natural history of HBoV2 infection is elucidated, the influence of S phase replication and asymptomatic shedding can only be speculative.

Although the data presented in Figure 4 suggests a close relationship between strains within each bocavirus species, it was evident from the failure of some primer pairs to amplify in the nested reaction that sequence variation exists in our primer binding sites. Further, while little trouble was experienced recovering the near identical genomic sequence of HBoV2-W208 using HBoV2-W153-derived primer sets, limited attempts with the more variant HBoV2 and HBoV3 strains has proved problematic. Therefore, sub-optimal primer binding may have led to an under representation of HBoV2 and HBoV3 in our samples. Indeed, Kapoor *et al.* [35] have recently reported a number of such variants. Alternate primers to improve the broad reactivity of the PCR assay, and real-time assays, are being investigated in a larger series of cases.

Until a quantitative assay is available, the viral load in both cases and controls cannot be assessed. However, a limited sample dilution analysis with current assays, for which the sensitivity has not been quantitatively assessed, have indicated viral loads range from 100 to at least 10^11^ DNA copies per gram of feces (data not shown).

Although our original isolation of HBoV2 was from a sample taken in the summer of 2001, screening of the 2001 sample set indicated HBoV2 was detected throughout the year from summer to early spring (Southern Hemisphere) with no obvious peak incidence. It is interesting to note that most AGE infections in Adelaide occur after this period, from late winter to early summer (predominantly rotavirus and NoVII). Additional screening is proceeding to assess if this distribution recurs annually.

Prevalence rates up to 8.6% have been reported for astrovirus [15]–[17] but the analyses were largely retrospective and uncontrolled. The Danish case control study by Olesen *et al*. did not find an association between astrovirus and AGE but the prevalence was low [7]. Dennehy *et al.* did report an association in a 5-year prospective case control study in hospitalized young children [39] but the analysis was flawed because controls were collected only during one quarter of the enrolment period and over 75% of the cases were unmatched. Thus our analysis confirming an association between astrovirus and AGE (prevalence 21.5%, P = 0.01, odds ratio 2.4, 95% confidence interval 1.2–5.0) is the first to do so in samples from matched case control pairs.

Recent reports have inferred HBoV may be associated with diarrhea in children [25],[26] but the studies were uncontrolled and retrospective. Co-infection with another agent was common. Further, when found in the stools of children with respiratory disease, detection correlated to high respiratory viral load [24]. Our analysis, which is the first to be based on fecal samples from prospectively enrolled cases and controls, does not find a association of HBoV with AGE. The lack of statistical association with symptoms is consistent with the presence of HBoV in feces being incidental, perhaps indicative of swallowed virus of respiratory origin [24] or systemic spread as has been reported for B19 parvovirus [36]. A recent prospective study supports our finding by detecting the asymptomatic acquisition of HBoV in respiratory secretions of healthy children less than one year of age [40].

In conclusion, a prospective case controlled study into the causes of AGE in children has identified a cluster of sporadic cases in whom conventional viral and microbiological analysis has failed to find a pathogen. Application of novel molecular testing techniques has resulted in the identification of one novel virus, HBoV2 with statistical data suggestive that it is causally associated with AGE and a second novel virus, HBoV3, with as yet no disease association. The detection of HBoV2 has reduced the pathogen-negative cases by 17.5%. Although not a prime focus of the study, we have additionally demonstrated a statistical association with AGE for astrovirus, the first such study to do so with prospectively enrolled matched case and control pairs. Lastly, we have similarly demonstrated that HBoV is not statistically associated with AGE and its presence in fecal samples is possibly the consequence of swallowing respiratory secretions. There is evidence of further genetic variation within strains which may be accounted for by additional variant strains of HBoV2 or HBoV and perhaps other bocaviruses. The role of HBoV3 in human disease is unknown. Although detected equally in samples from cases and controls, the prevalence is too low for meaningful analysis until further screening for its presence in a variety of samples has been completed.

Further investigation of the association of HBoV2 with AGE, and the role of HBoV3 in human disease, is required. We are continuing to test samples from over 600 cases and matched controls, and sequence additional bocavirus strains to determine the extent of sequence variation and to modify our screening assays accordingly. Confirmation of the presence of HBoV2 in AGE cases elsewhere in the world is needed as are further prospective controlled studies of viral prevalence in AGE. HBoV2 may be associated with diseases other than AGE and testing in other contexts will be needed. Finally, despite the addition of HBoV2 to the list of proven and probable causes of viral gastroenteritis, 25% of cases in this study remain undiagnosed. Additional viral causes of gastroenteritis may remain to be discovered.

## Materials and Methods

### Selection of faecal samples and screening for known infectious agents

Stool samples analysed in this study were collected from January to December 2001 from matched cases and controls who were part of a larger case control study into the aetiology of sporadic pediatric AGE. The study was approved by th je Research Ethics Committees of the Women's and Children's Hospital (WCH) and the Institute of Medical and Veterinary Science (IMVS). Following informed patient/parent/guardian consent, prospective enrolment was offered to patients presenting to the WCH in Adelaide, South Australia, and meeting the case definition of three or more loose stools within the last 24 hours, with or without vomiting, and a duration prior to presentation of less than 7 days. Clinical histories and parameters of disease progression were recorded for the enrolled cases. One or two faecal samples were collected from cases within 72 hours of enrolment but only a single sample from controls. Samples were screened for bacteria (microscopy and culture), parasites (microscopy) and viruses rotavirus and enteric adenovirus by antigen capture immunoassay [41] and caliciviruses and astrovirus by RT-PCR [17]. All PCR products were confirmed by nucleic acid sequencing before being classed as positive for the caliciviruses or astrovirus, see Table 1. In order to limit costs, the prevalence of bacteria and parasites, which have been previously assessed in prospective case control studies, was determined for the cases only, where the cost of testing was met by existing hospital pathology expenditure as part of patient management. Age matched controls (within 3 months for patients less than 6 months of age, within 9 months for patients 6 months to 2 years of age, within 12 months for 2 to 5 year old patients and within 24 months for patients older than 5 years) without symptoms of AGE were selected from patients and their siblings presenting to hospital at a similar time (median 8 days, maximum 35 days) with non-diarrheal conditions such as respiratory infections, asthma or surgical admissions. Insufficient controls were available to permit sex matching. Samples from an apparent cluster of undiagnosed sporadic AGE cases enrolled during the summer of 2001 were chosen for the initial screening for the presence of novel viruses since we hypothesised this may represent an outbreak of a novel viral infectious agent even though we had no epidemiological information to link the cases.

### DOP method

Stored frozen (−70°C) fecal samples were thawed and 0.5 ml of a 10% suspension in phosphate buffered saline was prepared for each and microfuged (13,000 rpm; 15 mins) to remove solids. Each supernatant was filtered through a 0.2 nm bacterial filter, digested with nucleases [30] and virions pelleted (28,000 rpm 2 hr, Beckman Coulter, Beverly, USA) before extracting the remaining viral nucleic acid fraction using the RNeasy Mini Kit system (QIAGEN, Hilden, Germany) according to the manufacturer's instructions. Nucleic acids not examined immediately were stored at −70°C. DOP PCR reactions were performed incorporating a single DOP primer and biphasic PCR amplification cycles [29],[32]. Briefly, the DOP 25-mer oligonucleotide primer consists of a 3′ four base anchor region which binds to both strands of any nucleic acid at approximately 250 base intervals. Adjacent to this is a six base degenerate region that provides sufficient binding homology to stabilize the primer binding at low annealing temperatures and permit priming and amplification during the first cycling phase which utilizes low annealing temperatures (30°C), slow temperature ramping and long extension times (5 minutes). In the initial replication cycles, the DOP primer 5′ tag region of 15 specific bases does not participate in the binding, but as replication proceeds, this sequence becomes incorporated at the termini of the amplified product, increasing the homology of the DOP primer to its target and therefore permitting the second cycling phase which utilizes higher annealing temperatures (50°C) and fast temperature ramping.

Gel analysis of the DOP amplification products was uninformative, revealing only a DNA smear. To monitor amplification efficiency, an adenovirus positive fecal sample was included in each batch of samples being amplified. If a 10^4^ or greater increase in adenovirus genome copies was detected in this sample, measured by a adenovirus-specific real-time PCR (unpublished), then the DOP-amplified products from the remaining samples in the batch were individually cloned without gel analysis into pCR TopoTA 4.0 vector and transformed into One Shot Topo10 competent cells (Invitrogen, Paisley, UK) according to manufacturer's instructions. Using sterile toothpicks, bacterial colonies were picked directly into PCR mix (25 µl) containing 1× GeneAmp PCR buffer (Applied Biosystems Inc [ABI], Foster City, USA), 2 mM MgCl_2_(ABI), 0.2 mM dNTPs (Finnzymes, Espoo, Finland), 1.25 U AmpliTaq Gold (ABI), and M13 universal forward and reverse primers (50 pmol each, Invitrogen) to amplify the DNA inserts. PCR products were assessed by gel electrophoresis and reactions containing single DOP amplicons of length greater than 250 nucleotides were sequenced with BigDye 3 terminator chemistry (ABI) using an ABI 3730 sequencing analyzer. Agencourt AMPure and Agencourt CleanSEQ (Beckman Coulter, Inc., Beverly, USA) were used to clean the PCR products before and after the sequencing reactions, respectively.

### Sequence analysis

DOP-generated sequence was screened for homology in GenBank (NCBI, Bethesda, USA) using tBLASTx (translated nucleotide versus translated nucleotide database) and BLASTn (nucleotide vs nucleotide database) searches. Sequence analysis, assembly of contiguous genome sequences, ORF analysis and sequence alignments throughout the study were performed using Kodon (Applied Maths, St-Martens-Latem, Belgium) incorporating the HBoV (strain st2, GenBank DQ000496) genome sequence as a comparison during sequence assembly. Gaps in the HBoV2 sequence were spanned using primers designed to the DOP-generated flanking sequences for PCR amplification from the original fecal specimen and sequencing. Distal sequences were generated using a semi-DOP approach whereby specific primers facing out to the termini of the HBoV2 genome, and the DOP primer, were used to amplify and sequence flanking DNA. The genome sequence was then rederived using specific overlapping primer sets spanning the genome to PCR amplify and sequence the viral DNA directly from the index fecal specimen (W153). Additional complete parvovirus sequences obtained from GenBank and used in the phylogenetic analyses (PAUP* 4, Sinauer Associates, Sunderland, Massachusetts) were as follows; NC_006148 snake parvovirus 1; NC_001701 goose parvovirus; NC_006147 Muscovy duck parvovirus; AF043303, AF063497, AF513851, AF513852, AF085716 Adeno-associated viruses; AY622943 human parvovirus 4; U86868 Chipmunk parvovirus; AF321230 Kilham rat virus; NC_001358 H-1 parvovirus; NC_001510 and DQ196317 minute virus of mice; NC_001630 mouse parvovirus 1; NC_001539 canine parvovirus; EF988660 Feline panleukopenia virus; NC_001718 porcine parvovirus; NC_001662 Aleutian mink disease parvovirus; AF221123 Pig-tailed macaque parvovirus; U26342 Simian parvovirus; NC_000883 human parvovirus B19; NC_006259 bovine parvovirus 2; DQ335247 and NC_001540 bovine parvovirus 1; AB158475 minute virus of canines and human bocavirus strains: St1 DQ000495; St2 DQ000496; WLL1 DQ778300; WLL2 EF441262; CU74 EF203922). The recently published sequence of HBoV2 (FJ170280) was also used in genome similarity comparisons.

### Nested consensus diagnostic PCR

DNA was extracted from 100 µl of a 10% (vol/vol) fecal specimen using the RNeasy Mini Kit system (QIAGEN) and amplified using a modification of the ‘hanging drop’ nested PCR method [17]. Current published primer sets to amplify HBoV [25],[26],[31],[32],[42] do not contain sufficient homology to successfully amplify products from HBoV2-W153. From an alignment of the NS1 gene sequences from HBoV2-W153 and HBoVs (DQ000495 and DQ000496), several primers (Sigma-Genosys, Castle Hill, Australia) targeting regions of shared sequence homology were chosen to amplify both HBoV2 and HBoV sequence. The primers were then tested in a variety of relevant combinations to ascertain which primer pairs achieved the most sensitive amplification in a nested PCR reaction, as measured by serial 10-fold dilutions of W153. Extracted nucleic acid (2.5 µl) was added to the primary amplification mix (25 µl) containing 1× GeneAmp PCR Buffer, 1.5 mM MgCl_2_, 0.2 mM of each dNTP 1.25 U AmpliTaq Gold and 10 pmol each of the primers Adel-OF (AGGTAAAACAAATATTGCAAAGGCCATAGTC) and Adel-OR (TGGGAGTTCTCTCCGTCCGTATC). The mix was overlaid with paraffin oil. The secondary amplification mix (25 ul) containing 1× GeneAmp PCR Buffer, 5.5 mM MgCl_2_ (to yield 3.5 mM during secondary amplification), 0.2 mM of each dNTP, 1.25 U AmpliTaq Gold and 40 pmol each of the primers Adel-IF (AGGGTTTGTCTTTAACGATTGCAGACAAC) and Adel-IR (TATACACAGAGTCGTCAGCACTATGAG), was located in the tube cap, to be suspended by surface tension during the primary amplification cycles. After 15 min activation at 94°C, 40 cycles of amplification (94°C for 30 secs, 52°C for 30 secs, 72°C for 1.5 min) were performed using an unheated lid. Once completed, the tubes were microfuged briefly to introduce the secondary amplification mix, and after 15 min activation at 94°C, 60 cycles of amplification as above, were performed. Several negative samples were included in each batch to monitor for false positive reactions resulting from contamination. Amplification products were visualized using agarose gel electrophoresis for products of the predicted size; primary amplification product, 732 bp, and secondary nested product 518 bp. Products of 590 and 659 bp may also be present, the result of amplification between an outer and inner primer pair. Products of the predicted size were sequenced using Big Dye 3 terminator chemistry (ABI) to confirm the sequence identity. Sequence alignments, error correction and similarity analysis (Maximum likelihood) were performed using Kodon and PAUP* 4. Additional sequence similarity analysis was performed with SimPlot 3.2 (S C. Ray, Johns Hopkins University, Baltimore, MD) [43].

### Statistical methods

A univariant analysis was performed on age-matched case and control pairs using McNemar's test for correlated proportions. A multivariant matched analysis was performed using conditional logistic regression. Calculations were carried out in VassarStats (R. Lowry Vassarstats, http://faculty.vassar.edu/lowry/VassarStats.html, Vassar College, Poughkeepsie, USA) and Stata version 9 (StataCorp, College Station, Texas).
